# Differential endopeptidase requirements during adaptation to changing growth conditions in Vibrio cholerae

**DOI:** 10.1099/mic.0.001671

**Published:** 2026-02-25

**Authors:** Kelly Rosch, Upasana Basu, Samantha Lei, Jennifer Zheng, Sven van Teeffelen, Tobias Dörr

**Affiliations:** 1 Weill Institute for Cell and Molecular Biology, Cornell University, Ithaca, USA; 2 Department of Molecular Biology and Genetics, Cornell University, Ithaca, USA; 3 College of Veterinary Medicine, Cornell University, Ithaca, USA; 4 Département de Microbiologie, Infectiologie, et Immunologie, Faculté de Médecine, Université de Montréal, Montréal, QC, Canada; 5 Department of Microbiology, Cornell University, Ithaca, USA

**Keywords:** beta-lactam, cell wall, endopeptidase (EP), hydrolase, osmolarity, peptidoglycan

## Abstract

The bacterial cell wall is a covalently linked meshwork of peptidoglycan (PG) that helps maintain cell shape and prevents osmotic lysis. This structure must be flexible enough to accommodate transenvelope protein complexes, but strong enough to withstand high intracellular pressure. To elongate and divide, cells must remodel the cell wall through the concerted action of PG synthesis and degradation. Endopeptidases, a class of PG-degrading enzymes, facilitate cell growth by hydrolysing PG crosslinks. *Vibrio cholerae* encodes several functionally redundant endopeptidases, two of which are nearly identical: ShyA and ShyC. To investigate the differential roles of these enzymes, we assessed the growth and morphology *of shyA* and *shyC* mutants. We found that native levels of ShyA, but not ShyC, facilitate adaptation to low-osmolarity medium. Cells lacking *shyA* exhibited a longer lag phase and aberrant morphology during adaptation. Lastly, our experiments revealed that cells lacking ShyA’s LysM domain exhibited more severe defects than cells lacking *shyA* altogether, implicating the LysM domain in the proper regulation of ShyA activity.

## Introduction

Nearly all bacteria contain a peptidoglycan (PG) cell wall, a covalently closed meshwork that establishes cell shape and protects the cell from osmotic lysis. PG consists of glycan strands, comprising polymerized alternating *N*-acetylmuramic acid and *N*-acetylglucosamine sugars, which are connected by peptide crosslinks [1,2]. In Gram-negative bacteria, the cell wall is a thin layer of PG enclosed between the inner and outer membranes in a space called the periplasm [3]. This thin PG layer must be malleable enough to accommodate the insertion of transenvelope protein complexes [4], while also being strong enough to withstand intracellular pressure roughly equivalent to that of a car tyre [2,5, 6]. To elongate and divide, cells must remodel PG without losing structural integrity. Cell wall remodelling requires the activity of PG synthases, which insert new PG material into the existing cell wall [7], and autolysins, a divergent group of enzymes that mediate cell wall turnover and create holes in the cell wall for new PG to be inserted [4,8, 9]. The activity of cell wall synthases and hydrolases is presumably tightly regulated because an imbalance in either activity can lead to severe growth defects or lysis [10]. Indeed, the activity of autolysins is a major contributor to the antibacterial mechanism of the β-lactam antibiotics, which are among the most widely prescribed antibiotics worldwide. β-Lactams inhibit cell wall synthases, resulting in autolysin-mediated cell wall degradation, which causes either cell death and lysis, or inhibition of cell division [11]. However, the mechanism by which this balance is maintained remains unclear.

Bacteria frequently encounter changing environmental conditions during their life cycles, which challenge the integrity of the cell wall and can disrupt the balance between these opposing classes of cell wall remodelling enzymes. *Vibrio cholerae*, the model organism in this study, lives in brackish water, pond water and the human gut, where it causes cholera disease [12]. During its life cycle, *V. cholerae* must adapt to changes in osmotic conditions and accommodate the resulting fluctuations in turgor pressure. As cells transition between different environments, substantial PG remodelling is likely required, further challenging the balance among PG remodelling enzymes.

Differential expression of specialized autolysins has been shown to play a role during environmental adaptation. Indeed, multiple carboxypeptidases and lytic transglycosylases have been shown to respond to changes in pH or outer membrane stress [13]. In *Escherichia coli*, the endopeptidases (EPs) MepS and MepM are only required under certain nutrient availability conditions [14]. In *V. cholerae*, the EP ShyB is produced only during zinc starvation [15]. Additionally, EPs may play a role in cell envelope modifications during mechanical stress; in *V. cholerae*, mechanical perturbations of the cell wall activate the VxrAB cell wall stress response system in a ShyA-dependent manner [16].

EPs are relatively uncharacterized compared with PG synthases, partially due to their functional redundancy, which makes simple genotype–phenotype dissection challenging. *V. cholerae* encodes nine EPs, many of which remain functionally uncharacterized. Two of these EPs are collectively required for normal growth: ShyA and ShyC, which are homologous to the *E. coli* d,d-endopeptidase MepM [8]. ShyA and ShyC are nearly identical; both contain an M23 metalloendopeptidase domain [17] and a LysM carbohydrate-binding domain [18]. ShyA is likely soluble in the periplasm, while ShyC is tethered to the inner membrane. ShyA and ShyC are conditionally essential [9]. While both EPs can fulfil normal growth functions in standard laboratory media, the reason for this apparent functional redundancy is unknown. Here, we assessed the roles of *V. cholerae* EPs ShyA and ShyC during adaptation to low osmolarity and found that EP activity is required for normal adaptation to salt-free medium, likely by buffering turgor-mediated cell expansion via PG cleavage.

## Methods

### Bacterial growth conditions

Liquid cultures were grown by shaking at 30 °C in Luria Bertani (LB) medium (LB Miller, Fisher Bioreagents #BP97235) or salt-free LB medium (SF-LB; tryptone 10 g l^−1^, yeast extract 5 g l^−1^). Where applicable, antibiotics were used at the following concentrations: streptomycin, 200 µg ml^−1^; carbenicillin, 100 µg ml^−1^. 5-Bromo-4-chloro-3-indolyl-β-d-galactopyranoside (X-Gal; 120 µg ml^−1^) was added to plates for blue–white screening, and sucrose (10%) was added to plates for counterselection against suicide vectors.

### Plasmid and strain construction

Plasmids were built using isothermal assembly [19] with primers listed in Table S1, available in the online Supplementary Material. Gene deletions were performed via homologous recombination using the suicide vector pCVD442 [20]. Chromosomal insertions were constructed using the vector pTD101, which inserts genes via double crossover into native *lacZ*.

All strains used in this study (Table S2) are derivatives of *V. cholerae* El Tor N16961 (WT). Plasmids were constructed and stored in *DH5α* and conjugated into *V. cholerae* using *E. coli SM10* or *MFD* donor strains. Liquid cultures of the donor strain were prepared in the appropriate antibiotic and mixed in an equal ratio (10+10 µl) with the recipient strain on an LB plate. After overnight incubation at 37 °C, cells were plated on LB containing streptomycin and carbenicillin to select for transconjugants. Colonies containing integration vectors were cured through two rounds of purification on salt-free sucrose agar containing streptomycin. Insertions and deletions were verified using PCR screening.

The *shyA::shyA*^∆^*^lysM^* strain was created by PCR-amplifying sequences upstream and downstream of the annotated LysM domain using primers DLP213/214 (upstream homology) and DLP215/216 (downstream). Fragments were column purified and cloned into Sma1-digested pCVD442 using isothermal assembly [19]. Deletion of *shyC* in this background was performed using pCVD∆*shyC* [9]. All strains were validated by whole-genome sequencing.

### BADA and FM4-64 staining

Strains were inoculated from frozen stocks into 5 ml of LB and incubated overnight at 30 °C with shaking. After incubation, cells were diluted 1:1,000 into LB or SF-LB. After 1.5 h, BADA was added to a final concentration of 125 µM. After 45 min of shaking incubation at 37 °C, cells were harvested and imaged on an agarose pad (0.8% agarose in LB or SF-LB) containing FM4-64 (5 µg ml^−1^). Cells were imaged using a Leica DMi8 inverted microscope, and the resulting images were processed minimally using Leica LasX software.

### Time-lapse microscopy

Cells were imaged under phase contrast on an agarose pad (0.8% agarose in LB or SF-LB) using a Leica DMi8 inverted microscope, with frames taken every 5 min. Stage temperature was set to 37 °C using a PECON TempController 2000-1.

### Quantitative image analysis

Time-lapse images were analysed using SuperSegger [21], and the resulting values were analysed and graphed using custom R scripts. Time to division was calculated by subtracting the frame in which each cell was first detected by SuperSegger (‘birth frame’) from the frame in which the cell divided, then multiplying that value by 5 to account for the number of minutes between frames. Maximum width was analysed separately using cell segmentation from Omnipose [22,23], followed by centreline-finding and local width determination using the ‘Pill Mesh’ option of Morphometrics [24], then finding the maximum value along the length of each cell.

### Growth curve analysis

Strains were grown overnight in LB and diluted 1:1,000 into LB or SF-LB in a 100-well honeycomb plate. The growth of each well was monitored by OD at 600 nm (OD_600_) on a Bioscreen C plate reader (Growth Curves America).

## Results

**Fig. 1.**
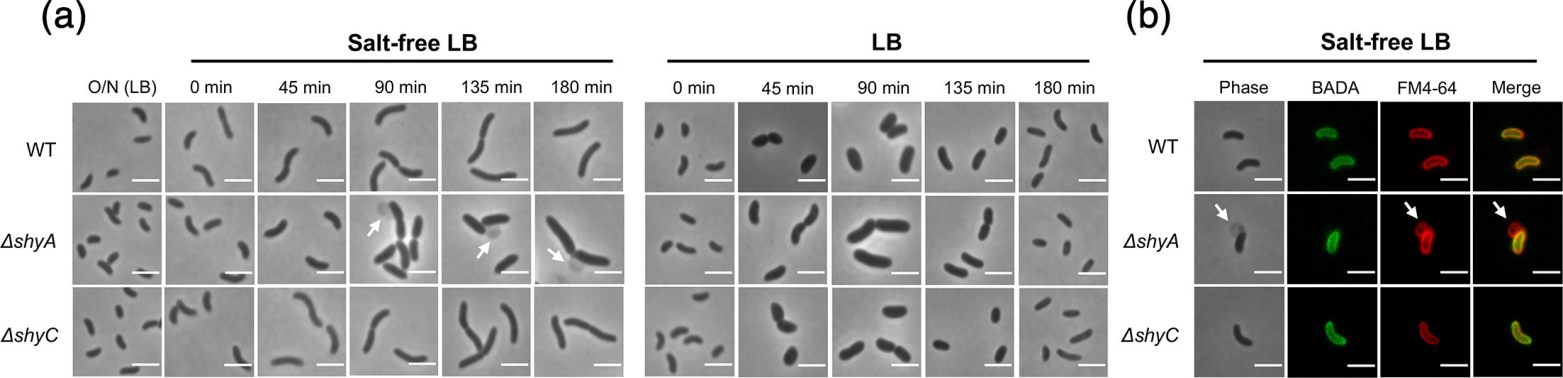
Cells lacking *shyA* exhibit a morphology defect in low-osmolarity medium. (**a**) After overnight incubation in LB, cultures were diluted 1:1,000 into LB or SF-LB, incubated at 37 °C, and imaged on an agarose pad (0.8%) at the indicated timepoints. Scale bar=3 µm. (**b**) BADA and FM4-64 staining after a 135 min incubation in SF-LB medium. Scale bar=3 µm. O/N = overnight culture.

*V. cholerae* encodes two principal EPs, ShyA and ShyC, which are constitutively expressed during normal growth. ShyA and ShyC are collectively essential, but individually redundant, like most autolysins in most bacteria [9]. We previously found that ShyA activity is required for *V. cholerae’s* ability to sense mechanical stress (such as strain experienced during mechanical compression or hydrostatic pressure) [16], and we, therefore, hypothesized that EPs may play a role in adjusting PG cleavage during changes in mechanical deformation of the cell envelope. A common factor deforming the cell envelope during an average free-living bacterium’s life is changing turgor pressure, e.g. during transition between growth environments with different ionic strengths. We thus investigated the requirement for ShyA and ShyC to ensure proper growth and morphogenesis during adaptation to low osmolarity (which results in increased turgor). To this end, we grew ∆*shyA* and ∆*shyC* mutants in a standard laboratory growth medium with high osmolarity (LB, 180 mM NaCl) or low osmolarity (SF-LB, 0 mM NaCl), and observed cell morphology (Fig. 1a). In LB, both mutants exhibited WT morphology. In contrast, in SF-LB, cells lacking *shyA* formed aberrant shapes, often with phase-light membrane blebs (Fig. 1a), while cells lacking *shyC* exhibited normal morphology under these conditions (Fig. 1a), indicating differential roles for EPs under these conditions. We next used the PG label BADA [25] and the membrane stain FM4-64to differentially visualize membranes versus cell wall. The blebs produced in the *∆shyA* mutant were stained with FM4-64, but contained qualitatively reduced PG labelling (BADA) compared with the cell body, indicating that this aberrant structure consists primarily of membrane material (Fig. 1b). These results may suggest a lack of PG expansion in the *shyA* mutant, which causes an imbalance in the production of cell envelope components; this is also reminiscent of the large outer membrane blebs we previously observed in strains lacking EP activity entirely [9,26].

**Fig. 2.**
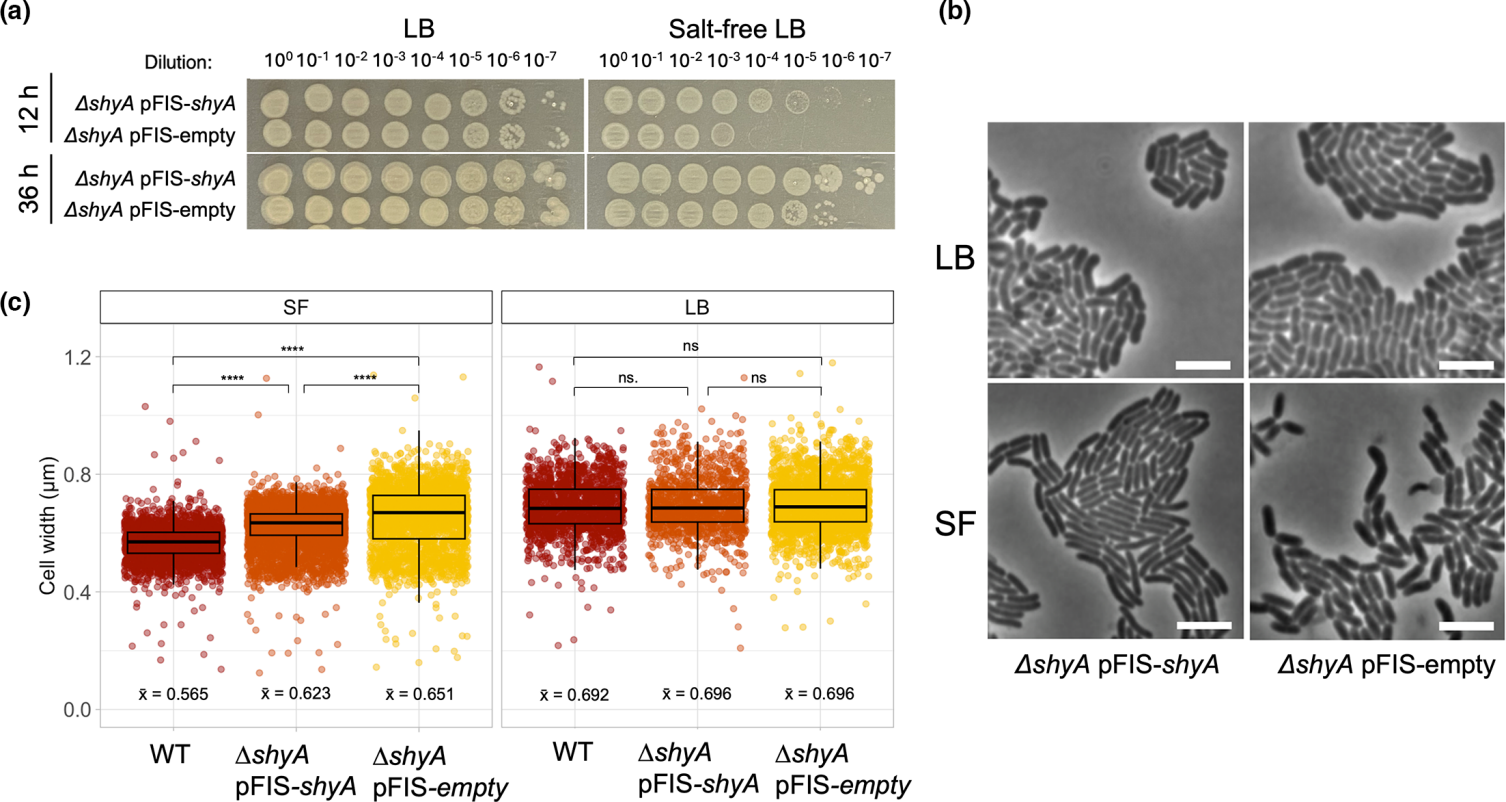
Cells lacking *shyA* exhibit transient growth and morphological defects on salt-free medium. (**a**) Spot dilutions of ShyA mutants were incubated at 37 °C on LB or SF-LB and imaged after 12 and 36 h. (**b**) ShyA mutants were imaged for 2.5 h using time-lapse microscopy on a 0.8% agarose pad containing LB or SF-LB. Scale bar=5 µm. (**c**) Cell length and width were quantified using SuperSegger; *P*-values were produced by pairwise comparisons using a linear model. ****P<0.0001. ns, not significant.

This single-cell phenotype also translated into population-wide growth delay upon plating: cells lacking *shyA* exhibited a slight but reproducible growth defect when plated on SF-LB but not on LB (Fig. 2a), which could be complemented by expressing *shyA in trans*. This growth defect on low-osmolarity medium became less pronounced after extended incubation, indicating that damage primarily occurs during the initial stages of adaptation to SF-LB medium (Fig. 2a). Cells lacking *shyC* did not exhibit a growth defect on LB or SF-LB (Fig. S1A); however, overexpression of ShyC rescued the *ΔshyA* growth defect on SF-LB, as did overexpression of the ShyA paralog ShyB, as well as a ShyC variant rendered soluble in the periplasm by replacing its transmembrane domain with a canonical DsbA signal sequence (Fig. S2). These data suggest that EP activity is important for adaptation to low-osmolarity environments, but ShyA may be either more active or more highly expressed during the transition. We noted that not all osmolytes rescued growth in *ΔshyA*. Cells lacking *shyA* were able to grow normally on SF-LB agar supplemented with 180 mM KCl, but not on SF-LB agar supplemented with 12.5% sucrose (the osmotic equivalent of 180 mM NaCl) (Fig. S2B). Indeed, sucrose, especially at higher concentration (15%), exacerbated the growth defect of the ∆*shyA* mutant (Fig. S3), while the ∆*shyC* mutant was viable under these conditions. Interestingly, overexpression of *shyC* and *shyB* counteracted this toxicity, while the soluble periplasmic ShyC variant did not (Fig. S2). It is possible that ShyA’s activity ensures proper cell wall homeostasis under both high- and low-tension states of the cell wall (induced by SF-LB=high turgor, or sucrose=low turgor, respectively); these data further suggest that ShyC’s membrane tether is important for activity under specific circumstances (such as sucrose exposure).

Next, we sought to quantify the adaptation dynamics to SF-LB at the single-cell level. To this end, we conducted time-lapse microscopy upon transitioning into a low-salt environment. While cells lacking *shyA* or expressing *shyA in trans* formed normal microcolonies on an LB agarose pad, the SF-LB condition induced a marked growth defect (Fig. 2b) specifically in *ΔshyA*. Cells lacking *shyA* exhibited a similar blebbing phenotype as observed in liquid medium, combined with qualitatively delayed growth, and consequently smaller microcolony formation (Fig. 2a, b). Cells lacking *shyA* were also significantly wider under these conditions than both WT and cells expressing *shyA in trans* (Fig. 2c), while on LB medium, none of the strains varied significantly in width (Fig. 2c).

**Fig. 3.**
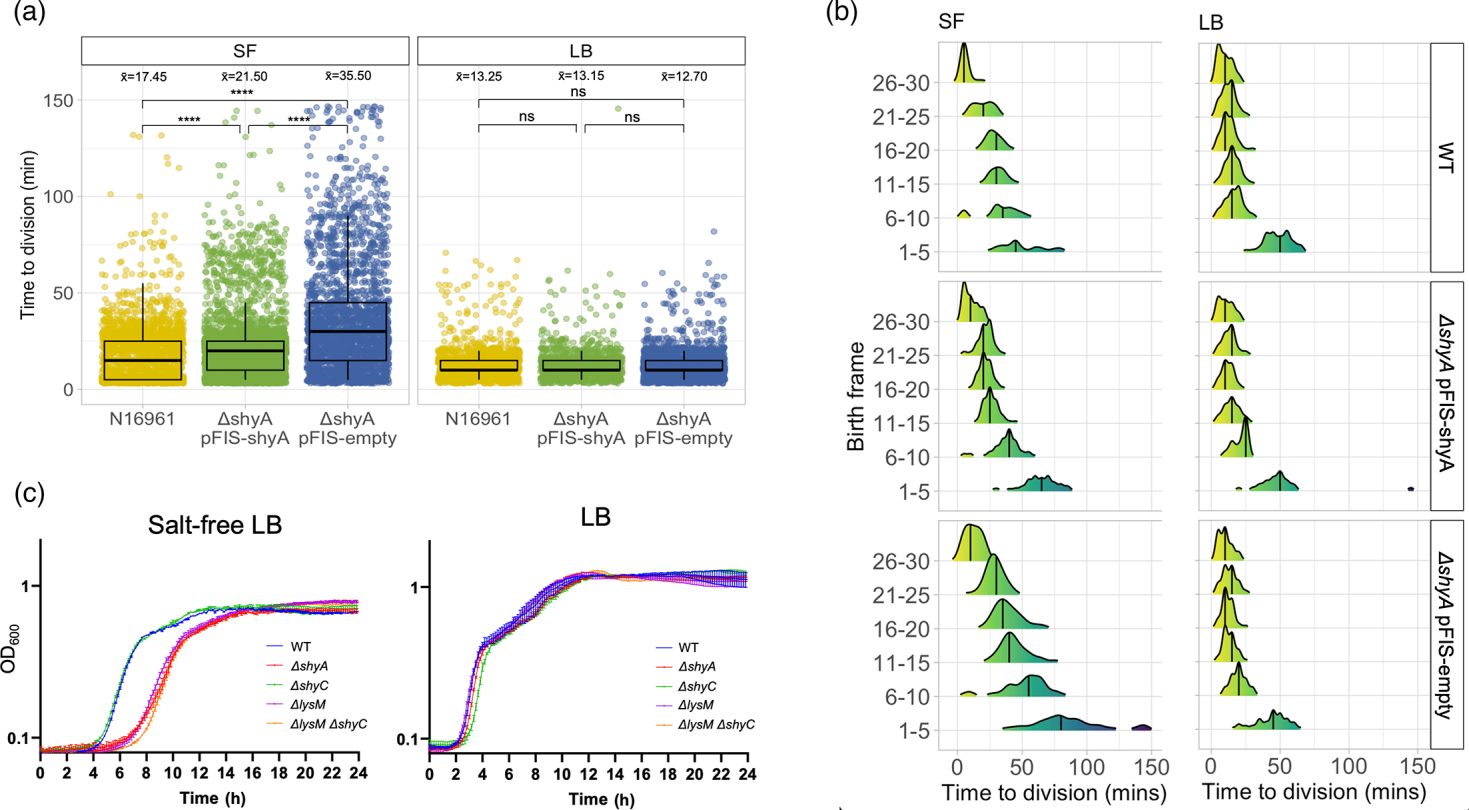
*shyA* mutants exhibit increased time to division on SF-LB. (**a**) Time to division was quantified using SuperSegger; each point represents one cell. (**b**) Time to division was binned by birth frame (the frame in which the cell was first detected by SuperSegger); frames were taken every 5 min. (**c**) OD_600_ measurements were taken every 10 min during incubation at 37 °C. ns, not significant.

The smaller microcolonies on the SF-LB agarose pad indicated that *ΔshyA* cells divided more slowly than WT. To quantify this, we analysed time-lapse images using SuperSegger [21], a software that enables lineage tracking for individual cells. Indeed, on SF-LB medium, cells lacking *shyA* took significantly longer to divide (mean=35.5 min±28.9) than WT cells (17.4 min±15.2), or cells expressing *shyA in trans* (21.5 min±16.5) (Fig. 3a). Conversely, all strains divided normally (12–13 min) when applied to an agarose pad containing LB medium (Fig. 3a).

 We then sought to distinguish adaptation to low osmolarity, i.e. the early stages of growth after transition, from the ability to grow in SF-LB long term. To investigate this, we binned cells based on the time-lapse frame in which they were first detected by SuperSegger (‘birth frame’) to ask whether defects were more pronounced early in the time-lapse (adaptation-phase defect) or evenly distributed throughout the time-lapse (overall growth defect). Indeed, on SF-LB medium, the division time for cells lacking *shyA* was longest during the early frames of the time-lapse and shortened as the time-lapse progressed (Fig. 3b), suggesting that ultimately, the ∆*shyA* mutant can adapt to low osmolarity. Conversely, on LB medium, the division time for all strains was slightly elevated during frames 1–5, presumably due to cells exiting lag phase, and then remained short for the duration of the time-lapse (Fig. 3b). Consistent with the single-cell observations, bulk populations of cells lacking *shyA* took longer to adapt and to grow in liquid SF-LB medium than cells expressing ShyA, while in LB, cells lacking *shyA* had no disadvantage (Fig. 3c). Together, these data indicate that the *ΔshyA* growth defect in SF-LB medium is most pronounced during the initial stages of adaptation to low osmolarity.

**Fig. 4.**
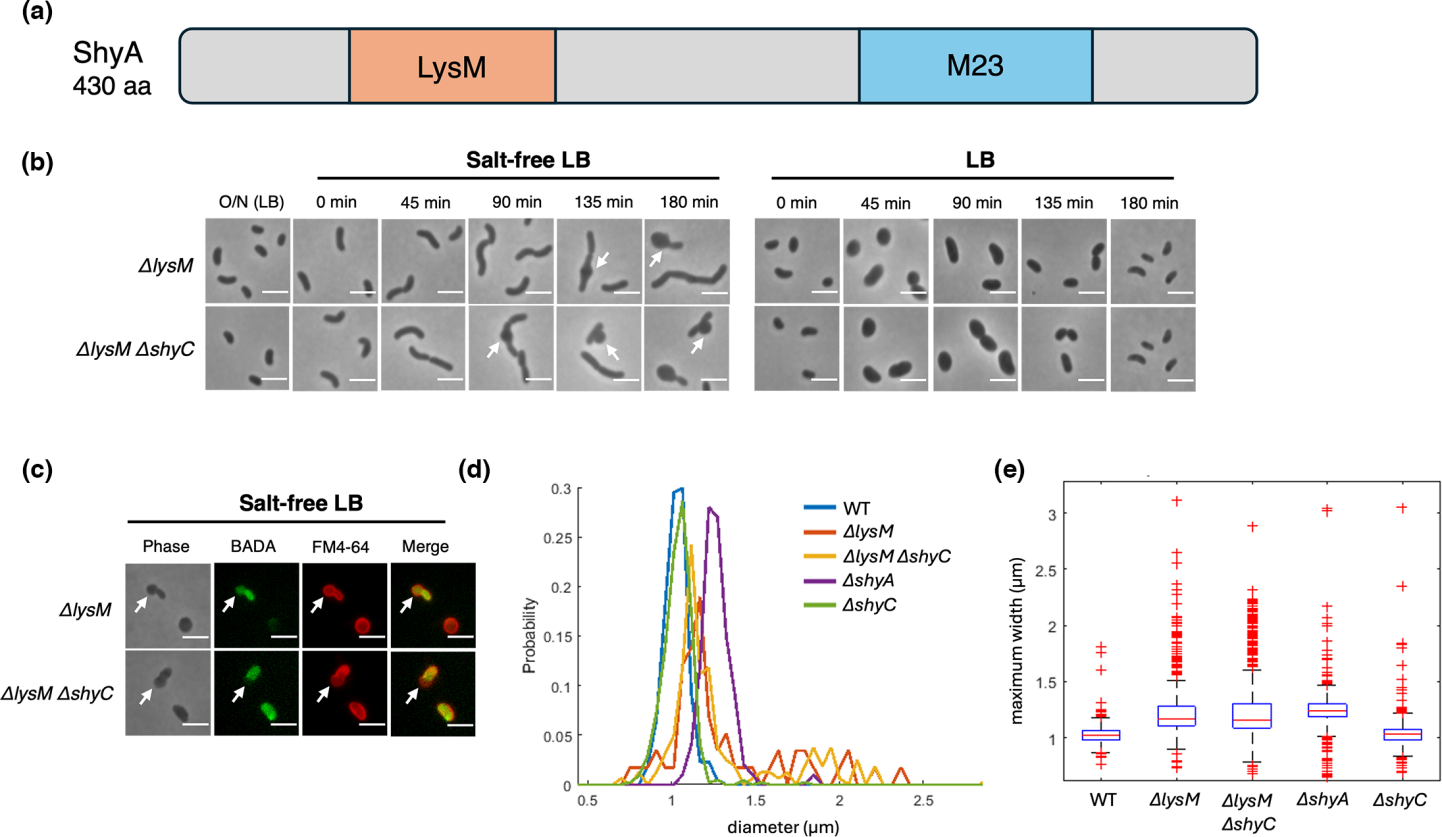
*shyA* phenotypes in SF-LB are dependent on the LysM domain. (**a**) ShyA contains a LysM carbohydrate-binding domain in addition to its M23 metalloendopeptidase domain. (**b**) After overnight incubation in LB, cultures were diluted 1:1,000 into LB or SF-LB, incubated at 37 °C and imaged on an agarose pad (0.8%) at the indicated timepoints. Scale bar=3 µm. (**c**) BADA and FM4-64 staining after a 135 min incubation in SF-LB medium. Scale bar=3 µm. (**d**) Cell diameter was measured using Omnipose and Oufti. (**e**) Maximum cell width (cell diameter +bulge width) was calculated using Omnipose and Oufti. aa, amino acids. O/N = overnight culture

The requirement for ShyA during adaptation indicated that perhaps ShyA needs to be more active under these conditions. ShyA exists in an open (active) conformation and a closed (inactive) conformation, in which the active site is blocked [27]. The mechanism for ShyA conformational switching remains unknown, but we proposed that allosteric interaction with ShyA’s substrate may promote opening. ShyA contains a LysM carbohydrate-binding domain that is predicted to bind to the glycan strands in PG (Fig. 4a) [18]. We thus reasoned that the LysM domain may play a role in adaptational activation during increased cell wall tension. To investigate this, we created a mutant lacking the LysM domain of ShyA (*shyA*^ΔlysM^). We were also able to construct a mutant lacking both ShyA’s LysM domain and ShyC (*shyA::shyA*^ΔlysM^ *ΔshyC*), demonstrating that ShyA^∆lysM^ is functional at least during growth in standard laboratory medium.

We then tracked the morphology of these mutants in LB and SF-LB. Like *ΔshyA*, both ∆*lysM* mutants exhibited morphology defects in SF-LB, but not in LB (Fig. 4b). Both *shyA*^ΔlysM^ strains showed bulbous protrusions, but unlike *ΔshyA*, these protrusions were phase dark instead of phase light, indicating that they contained cytoplasm. Cell wall staining (BADA) revealed that the protrusions contained qualitatively less BADA stain than the cell body, indicating that the balance between PG synthesis and degradation had been disturbed (Fig. 4c). Consistent with the *ΔshyA* defect, growth was also delayed in *shyA*^ΔlysM^ (Fig. 3c), and the cell width distribution of the *lysM* deletion mutant was shifted to a larger maximum diameter (in magnitude between ∆*shyA* and WT) (Fig. 4d, e). Thus, the LysM domain of ShyA is required for adaptation to low osmolarity, and the *shyA*^ΔlysM^ strain suffers from a dysregulated balance between PG degradation and synthesis during transition to lower osmolarity.

## Discussion

Here we show that the *V. cholerae* EP ShyA is important for adaptation to low osmolarity during transition out of stationary phase. While most studies on osmolarity challenges have focused on cytoplasmic solute homeostasis (e.g. cation exchange and compatible solutes [28,29]), we explored here the importance of EP activity in responding to osmotic stress on the cell wall. Cell wall remodelling may be a crucial and underappreciated component during bacterial life cycles as cells transition into new environments. Interestingly, ∆*shyA* cells adapting to low osmolarity resembled phenotypes observed during general EP insufficiency (outer-membrane blebbing [9,26]), suggesting that under these conditions, cell wall cleavage is a particularly limiting factor for optimal growth. As a side note, the outer-membrane blebbing we observed suggests that *V. cholerae* may not be able to modulate outer-membrane biosynthesis relative to cell wall biosynthesis when EP activity is limited.

 Cells encode multiple copies of each class of autolysins, indicating that these redundant genes may play differential roles under different conditions. Our work indicates a novel type of specialization for autolysins: an EP that optimizes adaptation to low osmolarity. The LysM carbohydrate-binding domain in ShyA appears to be critical for ShyA’s function under these conditions. While the role of the LysM domain in EP function is unknown, this domain is clearly central to ShyA activity, as cells without it perform worse than cells lacking *shyA* altogether.

 We have previously shown that ShyA activity is regulated by conformational switching between a closed (inactive) form and an open (active) form [27]. In the closed conformation, domain 1 of ShyA blocks the active site on domain 3. While the mechanism governing ShyA conformational switching remains unknown, we speculate that the LysM domain in ShyA domain 1 may bind PG at locations where crosslinks are under increased tension, leading to activation of ShyA and directing EP activity to locations on the cell wall where it is most needed. Though highly speculative, this idea would be consistent with the ‘smart autolysin’ hypothesis [30], and indeed our finding that ShyA becomes essential for adaptation to salt-free medium aligns with this idea. However, further experiments are needed to establish this idea.

## Supplementary material

10.1099/mic.0.001671 Uncited Supplementary Material 1.

10.1099/mic.0.001671 Uncited Supplementary Material 2.

## Abbreviations

EPs: endopeptidases
PG: peptidoglycan
SF-LB: salt-free LB medium
