# Supplementary material for: Differential endopeptidase requirements during adaptation to changing growth conditions in Vibrio cholerae
Source: Microbiology (Reading). 2026 Feb 25;172(2):001671. doi: 10.1099/mic.0.001671 (PMC13293342; doi:10.1099/mic.0.001671)
Supplement: Uncited Supplementary Material 1. [file mic-172-01671-s001.pdf]

Supplementary Figures

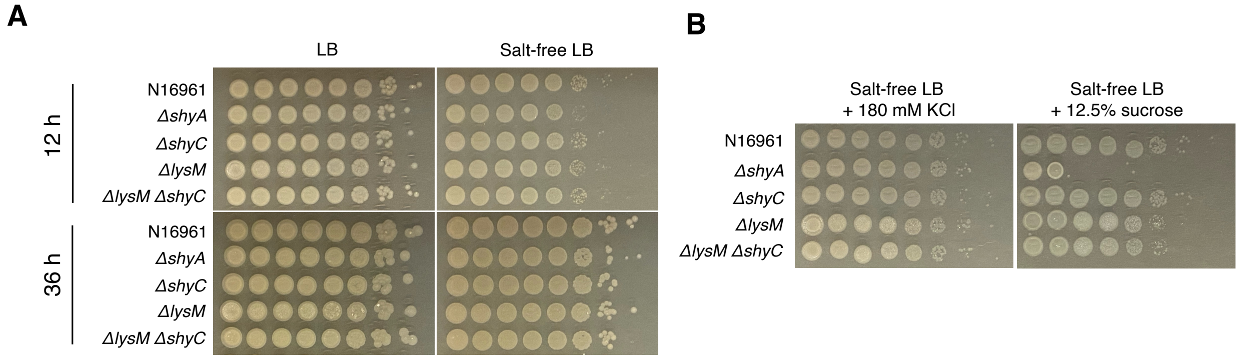

Figure S1. ShyC is not required for normal growth on low osmolarity medium and cannot fully rescue cells lacking *shyA*. (A) Spot dilutions of endopeptidase mutants were incubated at 37˚C on LB or SF-LB and imaged after 12 hours and 36 hours. **(B)** Spot dilutions were incubated at 30°C on SF-LB supplemented with 180 mM KCl or 10% sucrose and imaged after 12 hours.

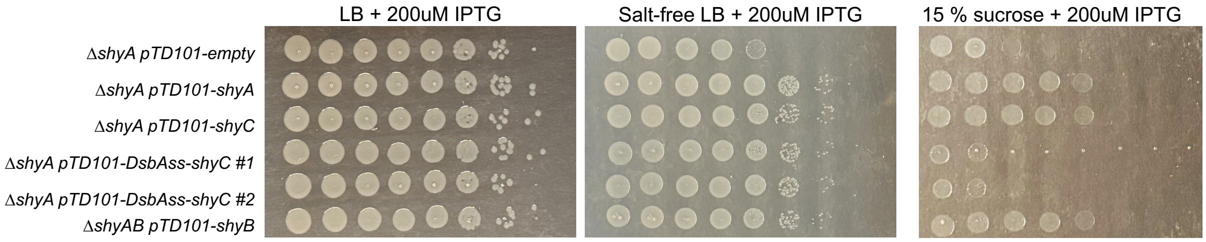

Figure S2 Overexpression of other endopeptidases can partially complement Δ*shyA* phenotypes. Cells were plates as described for Fig. 2S on LB agar with the indicated supplements.
